# Simulation of Groundwater Contaminant Transport at a Decommissioned Landfill Site—A Case Study, Tainan City, Taiwan

**DOI:** 10.3390/ijerph13050467

**Published:** 2016-05-04

**Authors:** Chao-Shi Chen, Chia-Huei Tu, Shih-Jen Chen, Cheng-Chung Chen

**Affiliations:** Department of Resources Engineering, National Cheng Kung University, Tainan City 701, Taiwan; chencs@mail.ncku.edu.tw (C.-S.C.); citizen0225@gmail.com (S.-J.C.); civilchn@ms21.hinet.net (C.-C.C.)

**Keywords:** GMS system, groundwater flow, contamination transport

## Abstract

Contaminant transport in subsurface water is the major pathway for contamination spread from contaminated sites to groundwater supplies, to remediate a contaminated site. The aim of this paper was to set up the groundwater contaminant transport model for the Wang-Tien landfill site, in southwestern Taiwan, which exhibits high contamination of soil and groundwater and therefore represents a potential threat for the adjacent Hsu-Hsian Creek. Groundwater Modeling System software, which is the most sophisticated groundwater modeling tool available today, was used to numerically model groundwater flow and contaminant transport. In the simulation, the total mass of pollutants in the aquifer increased by an average of 72% (65% for ammonium nitrogen and 79% for chloride) after 10 years. The simulation produced a plume of contaminated groundwater that extends 80 m in length and 20 m in depth northeastward from the landfill site. Although the results show that the concentrations of ammonium nitrogen and chlorides in most parts are low, they are 3.84 and 467 mg/L, respectively, in the adjacent Hsu-Hsian Creek.

## 1. Introduction

Generally, landfills will cause lots environmental pollution, such as smells in the air, landfill gas combustion, and wastewater leakage. Among all these, wastewater leakage affects the surrounding environment the most, especially the groundwater quality because wastewater leakage consists of high concentrations of organic compounds, heavy metal ions and toxic hazards. Recently, a large number of landfill-caused groundwater pollution cases have been reported around the world (Porowska [1], Sizirici and Tansel [2], Baker *et al.* [3], Peng *et al.* [4], Pleasant *et al.* [5], Han *et al.* [6], El-Salam and Abu-Zuid [7], Li *et al.* [8], and Zhou *et al.* [9]).

The contaminants of municipal solid waste derived from landfill leaks into groundwater aquifers because of rainfalls, and by groundwater flow they spread into the adjacent river system and pollute the ecosystem. However, this mechanism does not stop even after landfills have stopped receiving waste. Therefore, it is necessary to keep investigating and monitoring the surroundings of decommissioned landfill sites.

Today, many specialized computer software packages have been created and used to solve contaminant transport problems in groundwater system. However, Groundwater Modeling System (GMS), is the most powerful software package using the modular finite-difference flow model (MODFLOW), the particle-tracking post-processing model (MODPATH), the modular three-dimensional transport model (MT3DMS), the multi-species reactive transport model (RT3D), the finite element groundwater model (FEMWATER), the two-dimensional finite element model (SPEED2D), the sequential electron acceptor model (SEAM3D), the multi-phase flow transport model (UTCHEM), the general purpose parameter estimation (PEST), the various transport tools (UCODE) and the transition probability geostatistic software (T-PROGS) to work out groundwater contamination transport and the interaction between surface water and groundwater. The purpose of GMS is to predict the spreading of contaminant concentration by inputting initial conditions of hydraulic head, groundwater flow direction and the concentration of contaminant. Many studies have shown that the contamination transport in groundwater aquifers by using the GMS software package at the present time. (Brigham Young University [10], Abu-Rukah and Al-Kofahi [11], Al-Yaqout and Hamoda [12], Babiker *et al.* [13], Christensen *et al.* [14], Rapti-Caputo and Vaccaro [15], and Kim *et al.* [16]). As GMS is widely used to model groundwater flow and simulate contaminant transport in the world, thus in this paper, the authors decided to use GMS to perform the simulation work.

In this paper, in order to discuss the possibility of the land reusing in the decommissioned landfill site, Wang-Tien, the data of geology, hydraulic head, and contamination concentrations, *etc.* on landfill site were used in GMS to simulate and predict the contamination transport status. Decommissioned landfills are generally not protected the leachate spreading to underlying aquifers, which are required to be managed and controlled to avoid negative impact on the environment.

## 2. Methodology

Numerical method can effectively solve not only groundwater flow problem, but also contaminant transport of groundwater. By the rapid development of calculator technology, groundwater model has been applied to groundwater resource assessments, predictions and managements. GMS is an advanced and based on concept model groundwater aquifers simulation software, which provides lots of methodologies to establish groundwater flow numerical model. The following is the simulation process with explanations for each of the steps of this study.

### 2.1. Simulating Process

**Figure 1:**
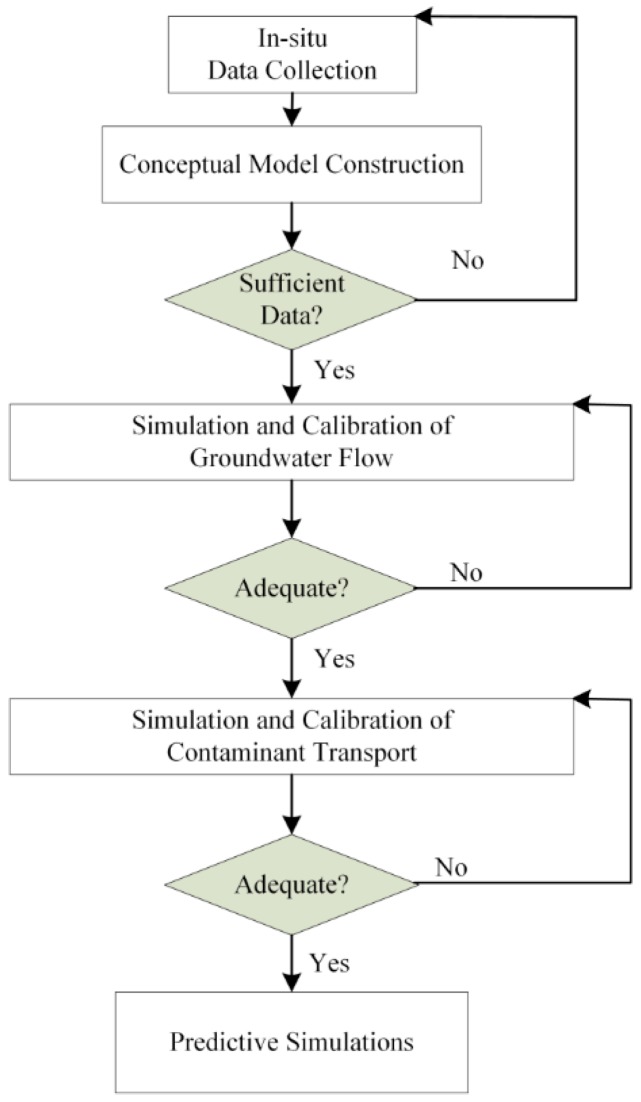
Flowchart of the modelling process.

The procedure for applying to a groundwater flow model includes the following steps (shown in Figure 1).

Step 1 Data Collection: During this step, *in-situ* information collection and measurement are necessary to collect information such as the annual rainfall and the annual evaporation from the Central Weather Bureau, Taiwan [17], and the geographic map from a geographic information system (GIS). Other data must be measured, such as geology, hydraulic head, groundwater flow velocity from slug tests of drilling bore-hole logs, and topographic elevation from topographic surveying, and groundwater sampling chemistry from the laboratory.

Step 2 Construction of a Conceptual Model: The conceptual model incorporates the information in Step 1 to establish a groundwater flow model which can be used to test and verify.

Step 3 Simulation and Calibration of Groundwater Flow Model: To perform the MODFLOW package to simulate groundwater flow on each month, then to calibrate the groundwater flow directions and the hydraulic heads until the simulation matches the *in-situ* observations to be optimized.

Step 4 Simulation and Calibration of the Contaminant Transport Model: After the groundwater flow model is calibrated, the contaminant source locations and contaminant concentration values are input into the model. Then the MT3DMS package is run to simulate the transport of contaminants in groundwater aquifer and subsequently to calibrate contaminant concentrations until the simulation matches *in-situ* observations to a reasonable degree.

Step 5 Predictive Simulations: After calibration, the model can be used to predict future groundwater flow and contaminant transport. The model may be used to estimate various remediation alternatives, such as risk assessment.

### 2.2. Governing Equations

The partial differential equations, describing the groundwater flow, velocity and contaminant transport, can be expressed as follows (Harbaugh, [18] and Zheng and Wang, [19]):

For the groundwater flow model:

(1) $$\frac{\partial }{\partial x}\left[k_{x}\left(\psi \right)\frac{\partial \psi }{\partial x}\right]+\frac{\partial }{\partial y}\left[k_{y}\left(\psi \right)\frac{\partial \psi }{\partial y}\right]+\frac{\partial }{\partial z}\left[k_{z}\left(\psi \right)\frac{\partial \psi }{\partial z}\right]=\left[\frac{\theta }{n}S_{s}+C\left(\psi \right)\right]\frac{\partial \psi }{\partial t}\pm Q\;$$

For the contaminant transport model:

(2) $$\frac{\partial }{\partial x}\left(\theta D_{xx}\frac{\partial C}{\partial x}+\theta D_{xy}\frac{\partial C}{\partial y}+\theta D_{xz}\frac{\partial C}{\partial z}\right)+\frac{\partial }{\partial y}\left(\theta D_{yx}\frac{\partial C}{\partial x}+\theta D_{yy}\frac{\partial C}{\partial y}+\theta D_{yz}\frac{\partial C}{\partial z}\right)+\frac{\partial }{\partial z}\left(\theta D_{zx}\frac{\partial C}{\partial x}+\theta D_{zy}\frac{\partial C}{\partial y}+\theta D_{zz}\frac{\partial C}{\partial z}\right)-\left[\frac{\partial }{\partial x}\left(\nu_{x}C\right)+\frac{\partial }{\partial y}\left(\nu_{y}C\right)+\frac{\partial }{\partial z}\left(\nu_{z}C\right)\right]=\frac{\partial }{\partial t}\left(\theta C\right)+\rho_{b}\frac{\partial s}{\partial t}+k_{m}\theta C^{m}\pm R$$

where *x*, *y*, *z* are the Cartesian coordinate axes; *t* is time; $k_{x},\;k_{y},\;k_{z}$ are the hydraulic conducitcity along the respective Cartesian *x, y*, and *z* coordinate axes; ψ is the pressure head; θ is the moisture content; n is the effective porosity of the porous media; $S_{s}$ is the specific storage of the porous media; C(ψ) is the specific moisture capacity; Q is a volumetric flux per unit volume representing sources and sinks of water; $D_{ij}\;\left(i,j=x,y,z\right)$ is the hydrodynamic dispersion coefficient; *C* is the concentration of contaminants dissolved in groundwater; $\nu_{x}$*,* $\nu_{y}$*,* $\nu_{z}$ are groundwater velocities in *x, y, z* directions and $\nu_{x}\;=\;-k_{x}\frac{\partial h}{\partial x}$*,* $\nu_{y}\;=\;-k_{y}\frac{\partial h}{\partial y}$*,* $\nu_{z}\;=\;-k_{z}\frac{\partial h}{\partial z}$*,* $h\;=\;z^{\prime }\;+\;\psi$ is the total head; $z^{\prime }$ is elevation head; $\rho_{b}$ is bulk dry density of the porous media; s is the weight of adsorbed water per unit area of porous media; $k_{m}$ is the decay coefficient of the contaminant concentration; m is the m^−th^ order of chemical/biological decay; *R* is the retardation coefficient, define as:

(3) $$R=1+\frac{\rho_{b}}{n}K_{d}$$

where $K_{d}$ is partition coefficient. The components of the hydrodynamic dispersion coefficients are also calculated by Equation (4):

(4) $$D_{xx}=\alpha_{L}\frac{\nu_{x}^{2}}{\left|\nu \right|}+\alpha_{TH}\frac{\nu_{y}^{2}}{\left|\nu \right|}+\alpha_{TV}\frac{\nu_{z}^{2}}{\left|\nu \right|}+D^{*}\;D_{yy}=\alpha_{L}\frac{\nu_{y}^{2}}{\left|\nu \right|}+\alpha_{TH}\frac{\nu_{x}^{2}}{\left|\nu \right|}+\alpha_{TV}\frac{\nu_{z}^{2}}{\left|\nu \right|}+D^{*}$$

where $\alpha_{L}$ is the longitudinal dispersivity; $\alpha_{TH}$ is the horizontal transverse dispersivity; $\alpha_{TV}$ is the vertical transverse dispersivity; $D^{*}$ is the effective molecular diffusion coefficient;$\;\left|\nu \right|=\sqrt{\nu_{x}^{2}+\nu_{y}^{2}+\nu_{z}^{2}}$ is the magnitude of the velocity vector. When the velocity vector is aligned with the same coordinate axes, all the cross terms become zero.

Those governing equations cannot be solved analytically, therefore, they have to be solved by using numerical methods. The finite difference method is used in MODFLOW and MT3DMS package for deriving the solution to the governing equation in this study.

## 3. Information on the Study Site *In-Situ*

### 3.1. Description of the Study Site

**Figure 2:**
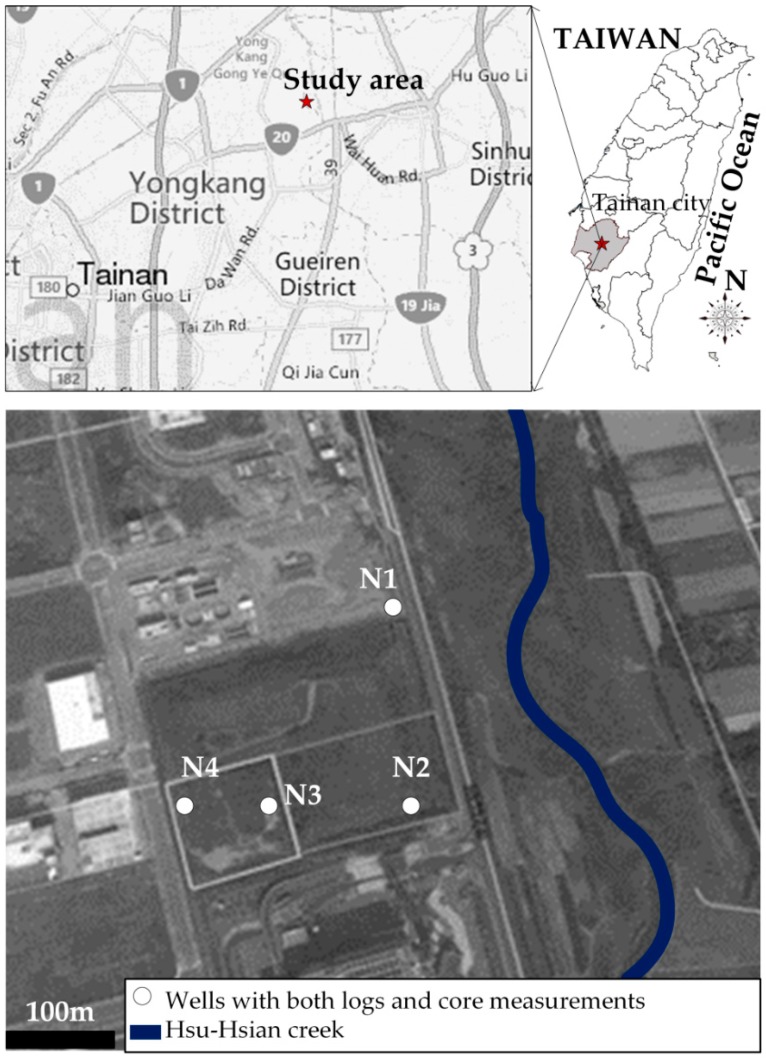
Location of study area.

The study site, the Wang-Tien landfill site, is located in the Yong-Kang Municipality of Tainan region as shown in Figure 2. The landfill, causing an accumulative amount of solid waste to reach approximately 773,970 m^3^, started to load municipal solid waste in 1992 and was decommissioned in 2002. The location of the study area lies between the longitudes 23°2′29′′ N and 23°2′36′′ N and latitudes 120°16′1′′ E and 120°16′22′′ E, and it occupies a total area of 39,333 m^2^. The elevation of the study area ranges between 8.75 and 25 m above mean sea level, and with a mean land surface slope of 0.1‰ from southeast to northwest. The landfill site encompasses an area 175 m north-south, and 225 m east-west, and is currently surrounded by Yong-Kang industrial zone. The site is located within the Hsu-Hsian Creek system which drains into the Yan-Shuei Creek. The mean value of precipitation in the Tainan area is 1828.4 mm/year and the mean value of the evaporation in the field area is 1476 mm/year.

**Figure 3:**
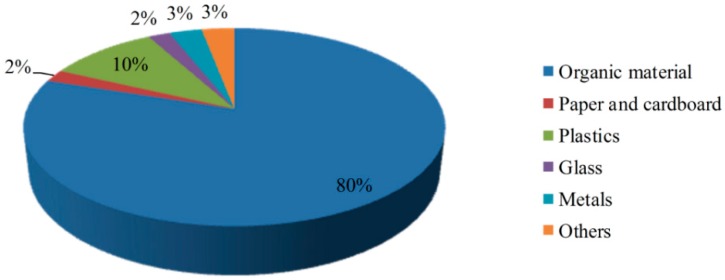
Composition of municipal solid waste in the Wang-Tien landfill site.

The composition of municipal solid waste in Wang-Tien landfill site is shown in Figure 3, where organic waste constitutes 80%, which was mostly household waste without separation or treatment.

### 3.2. In-Situ Data Collection and Measurement

**Figure 4:**
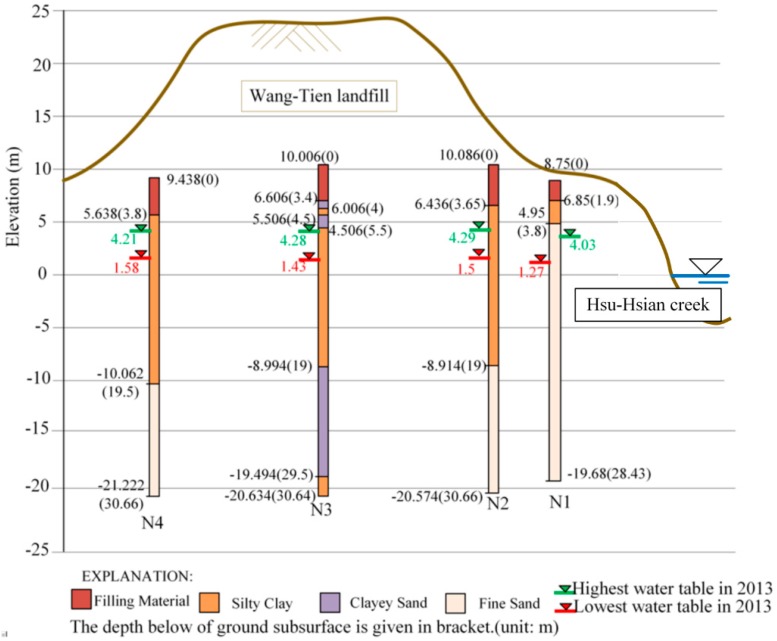
The major geological layers in the Wang-Tien landfill site.

There are four borehole loggings in Figure 4, showing the major geological layers in the study site. Unlike ordinary soil, the filling material layer, which thickness is about 3.8 m, is a mixture of municipal solid waste and grains of different sizes from clay to sand, concretes and gravels. After observing carefully the four bore-hole loggings, we found that the soil profile arrangement of the N3 located in the central landfill site is quite distinct from three others, because there is about 0.6 m thick clay sand layer starts at a depth 3.4 m below ground surface. The Wang-Tien landfill site is an older landfill without installing the lining, it was selected to be a landfill site might because the natural clayey sand layer of the ground subsurface can prevent leachate spreading to underlying aquifers.

**Figure 5:**
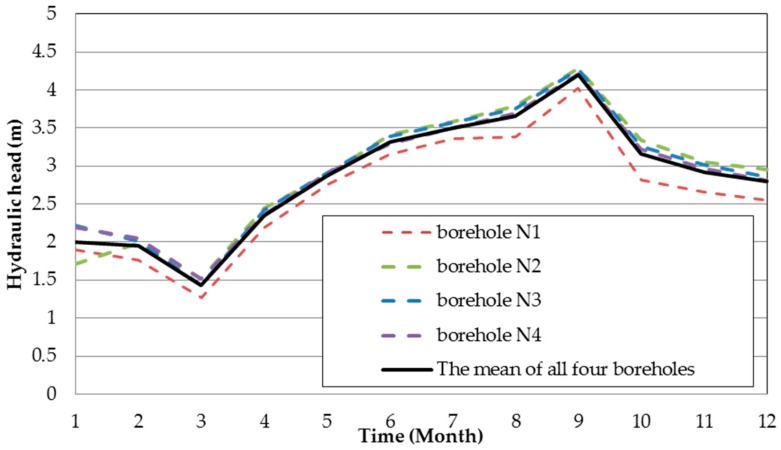
Annual hydraulic heads variation in 2013.

**Figure 6:**
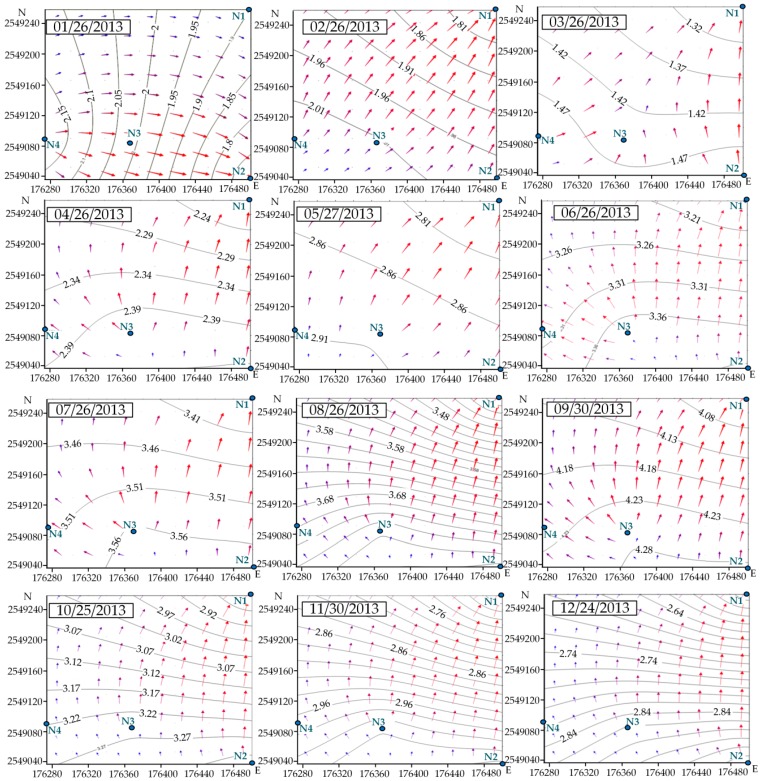
Hydraulic head contours maps: modeling with, Surfer v9, computer software (ordinary kriging interpolation). The arrows indicate the flow direction of groundwater.

The mean hydraulic head of all boreholes was at a depth of about 7 m below ground surface. The hydraulic heads derived from *in-situ* borehole data monthly measured in 2013 as shown in Table 1. According to the data of Table 1, the annual hydraulic head variations were plotted in Figure 5, and then combined the data of Table 1 with the locations of boreholes, the monthly hydraulic heads contour maps of 2013 were drawn in Figure 6.

The hydraulic conductivity data of each soil layer were taken from *in-situ* and laboratory hydraulic test as shown in Table 2. By the way, the hydraulic conductivity tensor in the numerical model was assumed to be isotropic.

In Table 3, it shows contaminant concentration data obtained from a laboratory sampling work carried out in November 2013 and the current standards from the Environmental Protection Agency (EPA in Taiwan [20], current standards given in brackets). According to those values of pollutants, we confirmed that groundwater was polluted and considered as serious risks. We also discovered in some parts of the site that contaminated the ammonium nitrogen and chlorides were over the Taiwan EPA standard. In addition, the pollutants are unevenly distributed. Accordingly, the contaminant of ammonium nitrogen and chlorides, exhibiting the higher concentrations, were chosen to simulate and discuss its fate and transport in this study.

**Figure 7:**
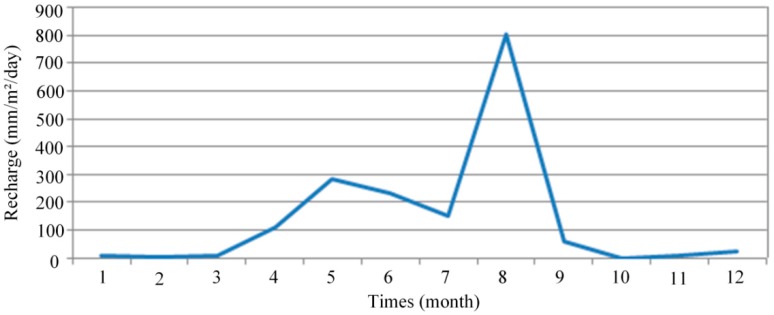
Transient recharge variation in 2013 (Each point on the curve was plotted by taking the open data of monthly measurements [17]).

Since monitoring rainfall data were not available in the Wang-Tien landfill area, the data for the Tainan weather station, close to Wang-Tien, should be the second best option. The rainfall data were from the website of Central Weather Bureau of Taiwan, which recorded rainfalls for each day, hour by hour, can be found from 1 January 2007, until today. From these data, rainfalls have been calculated for each month, in mm/m^2^/month first, then in mm/m^2^/day to correspond to the units used in GMS modeling. Figure 7 shows the recharge variation in 2013.

## 4. Numerical Simulation

GMS is a powerful simulator. A great quantity of input data for GMS is collected from a literature review, field investigations, and laboratory reports (Mehnert and Hensel, [21] and Bedient *et al.* [22]). In order to simulate and predict the contamination transport of the surroundings in the decommissioned landfill, Wang-Tien, the information on geology, topography, monthly rainfalls, the soil layers, various contamination concentrations and the monthly hydraulic head were taken from *in-situ* bore-holes as shown in Section 3.

In the numerical simulation, a correct description of hydrological and geological conditions at the study site is necessary which can be used to numerically model the groundwater flow and contaminant transport processes. In the conceptual model in which data describing site conditions are assembled in a systematic approach to indicate groundwater flow and simulate contaminant transport processes of the ammonium nitrogen and chlorides at the study site.

### 4.1. Numerical Model Construction

The numerical model of Wang-Tien landfill site was constructed by using package MODFLOW in this study. A rectilinear grid pattern was used to divide the model domain both horizontally and vertically into rectangular cells in package MODFLOW to calculate groundwater table conditions in each cell of the model domain.

**Figure 8:**
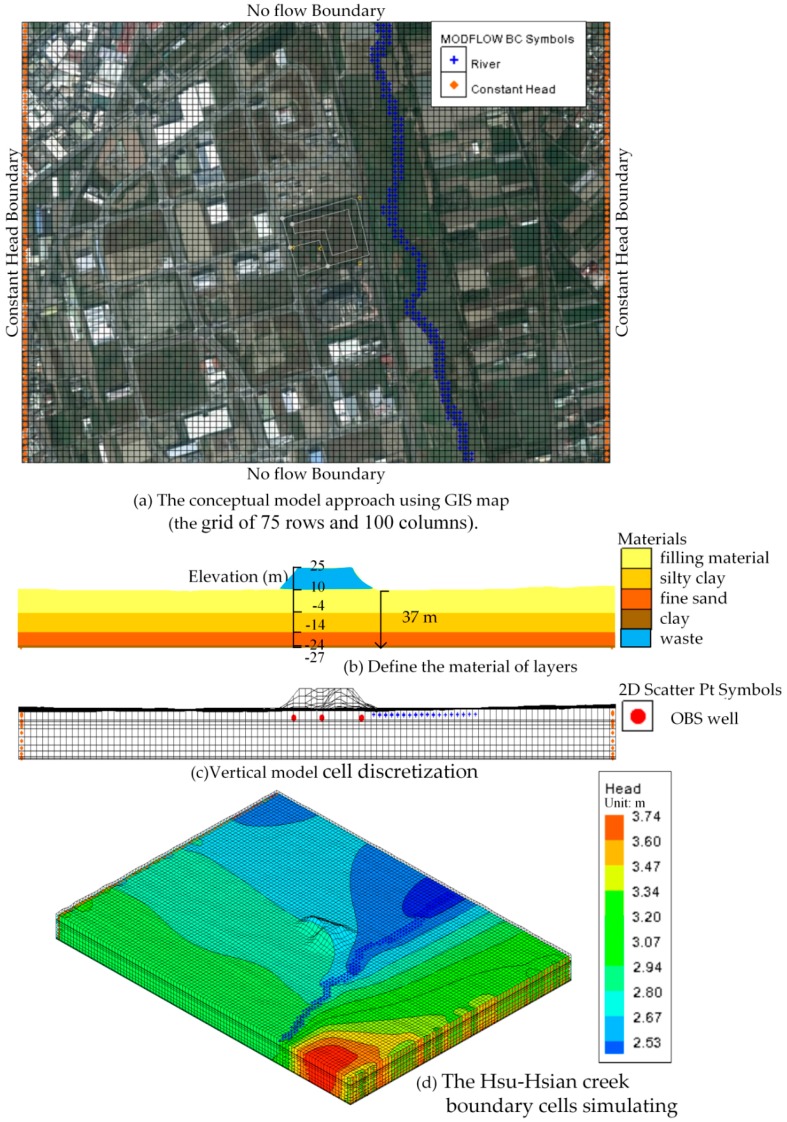
The MODFLOW flow model meshing. (**a**) The conceptual model approach using GIS map (the gird of 75 rows and 100 columns); (**b**) Define the material of layers; (**c**) Vertical model cell discretization; (**d**) The Hsu-Hsian creek boundary cells simulating.

To avoid an inaccurate simulation from the boundary effect, it is necessary to construct a larger grid model with boundaries far enough from the study site. Thus, the model domain boundary is extended about 750 m north-south and about 1000 m east-west and the model domain is discretized into a grid of 75 rows and 100 columns. Each layer in the numerical model contains 7500 grid cells as shown in Figure 8a. These cells are 20 by 20 m in the entire model domain. The vertical domain, about 37 m thick of variable elevation below ground surface, is discretized into 4 layers of varying thickness as shown in Figure 8b,c represented the vertical model domain with several approximations due to model cell discretization. The depth of the bottom boundary constraints on groundwater flow in the shallow units where the majority of contaminant transport congests. The model layer surfaces were developed by interpolation of borehole stratigraphy and ground surface topography, within a GIS.

### 4.2. Boundary Condition

The northern (downriver boundaries) and southern boundaries (upriver boundaries) of the model domain were represented as no flow boundaries, where the assumption that flow in these outlying areas was generally east-westward and parallel to the Hsu-Hsian creek, due to recharge in high elevation areas and discharge to Hsu-Hsian creek and the constant head boundaries (CHB) were applied to the eastern and western boundaries of the model domain. The values of the boundary hydraulic head, determined from the topographic survey data, were assigned to be a constant with the Hsu-Hsian creek elevations.

Average groundwater elevations in the study area are based on *in-situ* measurements from all monitoring boreholes (Figure 5). These boundary values were assigned to all layers of the model domain. The Hsu-Hsian creek was assigned in the model as river boundary cells simulating the hydraulic interaction between surface water and groundwater systems (Figure 8d).

According to the hydrological and the geological setting of the study site, an aquifer system is present. It is an unconfined aquifer of one homogenous fine sand layer. Its top is the filling material layer and its bottom is the clayey sand layer. The groundwater aquifer recharge is dependent on various factors, including the hydraulic conductivity, topography and amount of rainfall. In the numerical model of this study, a surface, the top layer boundary (filling material layer), above the groundwater flow was subjected to a transient recharge. The value of transient recharge was based on the variable data of the monthly rainfall in 2013 (Figure 7). 

**Figure 9:**
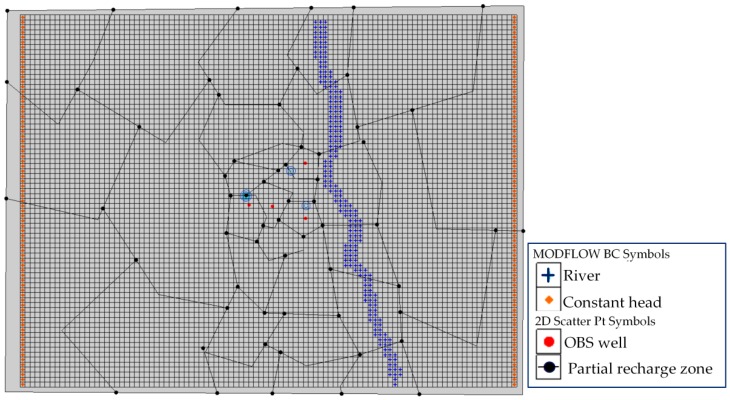
The recharge divisions.

In the numerical domain, the recharge surface was divided into 30 partial recharge zones, and each borehole was located at different zones as shown in Figure 9. These partial zones can be adjusted their recharge for calibration work. The mean value of precipitation in the Tainan area is 1828.4 mm/year and the mean value of the evaporation in the study area was assumed to be 1476 mm/year. Therefore, the recharge for the study site is 352.4 mm/year can be estimated.

### 4.3. Parameters Inputting

All the aquifer parameters, shown in Table 4, are assumed that the aquifer was contaminated by polluted waste water (containing the ammonium nitrogen and chloride) via several injected wells at the speed of 19.2 m^3^/day with the ammonium nitrogen concentration of 35 mg/L and the chloride concentration of 4240 mg/L for contaminant transport simulation. The dispersivity can be expressed as follows empirical equation (Wong and Hayduk, [23]):

(5) $$\alpha_{L}=0.1\times L_{d},\;\alpha_{T}=0.2\alpha_{L}$$

where $\alpha_{L}$ is longitudinal dispersivity; $\alpha_{T}$ is transverse dispersivity; $L_{d}$ is the highest elevation at the location of the contaminant source. The Wang-Tien landfill, located at the highest elevation was 25 m, was the main source consecrating contaminant in the study area. Thus, the longitudinal dispersivity $\alpha_{L}$ is taken as 2.5 m and transverse dispersivity transverse dispersivity of the horizontal $\alpha_{TH}$ and the vertical $\alpha_{TV}$ are taken as 0.5 m in this simulation.

## 5. Results and Discussion

In order to obtain the simulation result more in line with the *in-situ* conditions, we defined the one year groundwater flow model according to varying hydraulic heads with the monthly measurement data from 2013. There are 12 serial simulations indicating the groundwater flow situation for each month in this model, and the groundwater flow of those serial simulations is continuously via repeatedly failing the calibration process. Furthermore, the one year groundwater flow model was recorded and it can use to simulate and predict the contaminant transport in this study.

### 5.1. The One Year Groundwater Flow Model Simulation and Calibration

The calibration of the groundwater flow model, constructed in the most time-consuming effort within the modeling procedure, is a key to its applicability. The calibration procedure could be done manually by adjusting input variables to find an optimal scheme which is the closet agreement between the simulation and measurement. In this study a threshold value for the hydraulic head was equal to 2 m. The hydraulic head simulations were considered than ±2 m and the adjusting input variables were hydraulic conductivities and recharge rates changing in each partial recharge zone during the calibration process.

**Figure 10:**
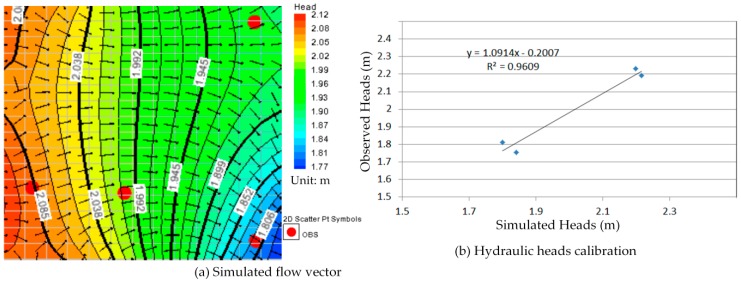
Groundwater flow vector and hydraulic heads calibration on 26 January 2013. (**a**) Simulated flow vector; (**b**) Hydraulic heads calibration.

**Figure 11:**
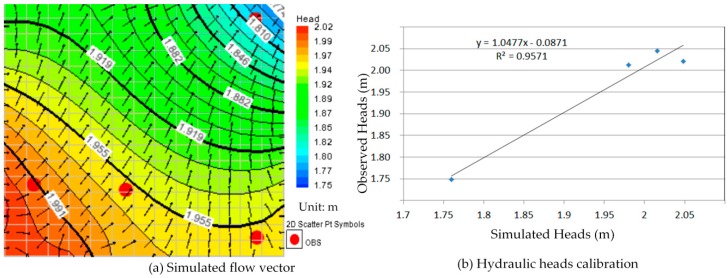
Groundwater flow vector and hydraulic heads calibration on 26 February 2013. (**a**) Simulated flow vector; (**b**) Hydraulic heads calibration.

**Figure 12:**
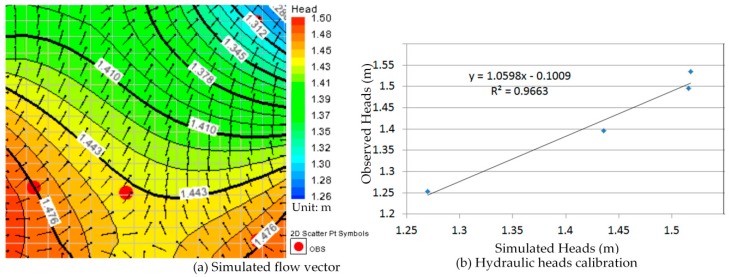
Groundwater flow vector and hydraulic heads calibration on 26 March 2013. (**a**) Simulated flow vector; (**b**) Hydraulic heads calibration.

**Figure 13:**
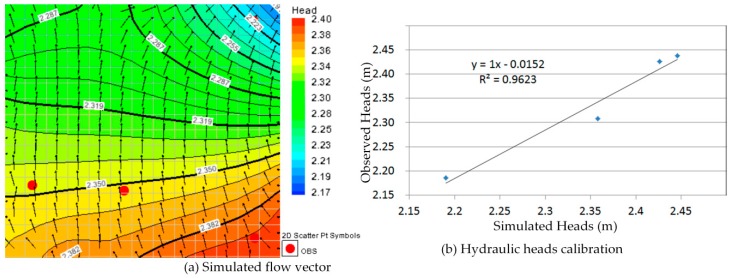
Groundwater flow vector and hydraulic heads calibration on 26 April 2013. (**a**) Simulated flow vector; (**b**) Hydraulic heads calibration.

**Figure 14:**
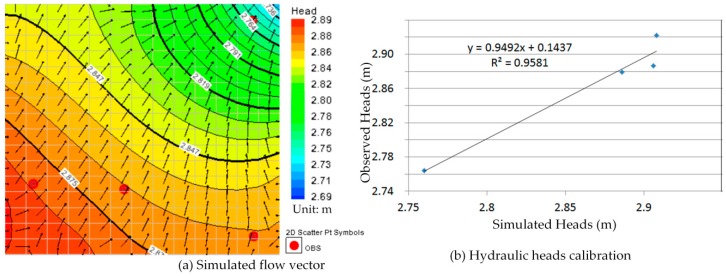
Groundwater flow vector and hydraulic heads calibration on 27 May 2013. (**a**) Simulated flow vector; (**b**) Hydraulic heads calibration.

**Figure 15:**
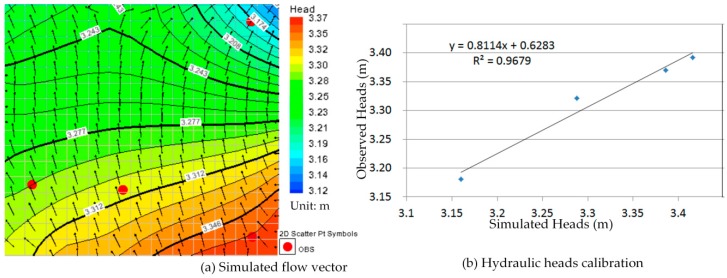
Groundwater flow vector and hydraulic heads calibration on 26 June 2013. (**a**) Simulated flow vector; (**b**) Hydraulic heads calibration.

**Figure 16:**
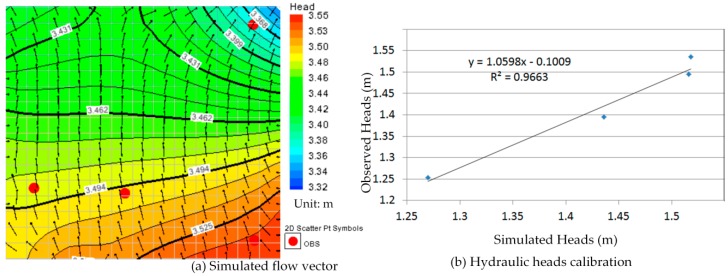
Groundwater flow vector and hydraulic heads calibration on 26 July 2013. (**a**) Simulated flow vector; (**b**) Hydraulic heads calibration.

**Figure 17:**
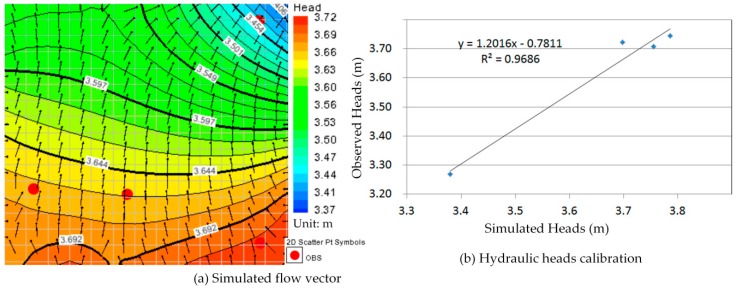
Groundwater flow vector and hydraulic heads calibration on 26 August 2013. (**a**) Simulated flow vector; (**b**) Hydraulic heads calibration.

**Figure 18:**
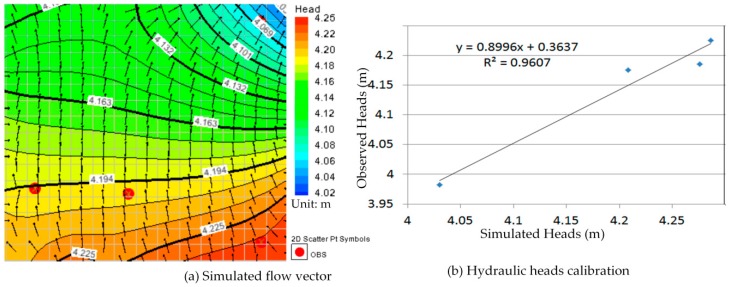
Groundwater flow vector and hydraulic heads calibration on 30 September 2013. (**a**) Simulated flow vector; (**b**) Hydraulic heads calibration.

**Figure 19:**
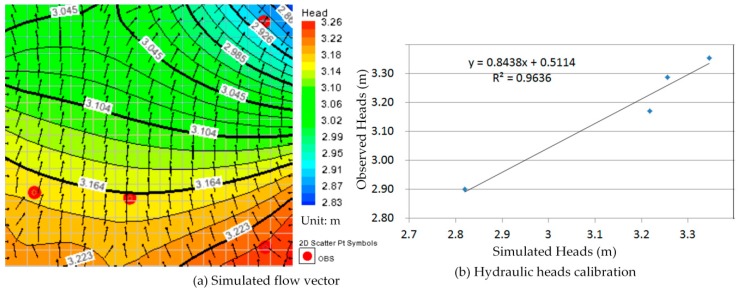
Groundwater flow vector and hydraulic heads calibration on 25 October 2013. (**a**) Simulated flow vector; (**b**) Hydraulic heads calibration.

**Figure 20:**
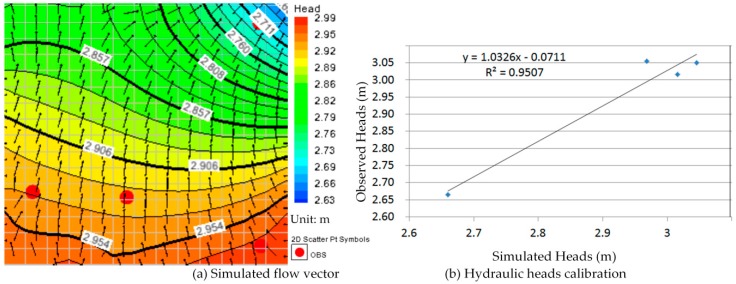
Groundwater flow vector and hydraulic heads calibration on 30 November 2013. (**a**) Simulated flow vector; (**b**) Hydraulic heads calibration.

**Figure 21:**
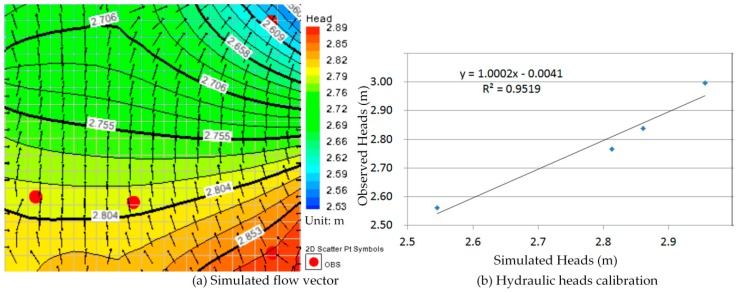
Groundwater flow vector and hydraulic heads calibration on 24 December 2013. (**a**) Simulated flow vector; (**b**) Hydraulic heads calibration.

Comparison between Figure 10a, Figure 11a, Figure 12a, Figure 13a, Figure 14a, Figure 15a, Figure 16a, Figure 17a, Figure 18a, Figure 19a, Figure 20a and Figure 21a show that the groundwater flow vectors almost all point to the adjacent Hsu-Hsian creek, and compared with the Figure 6 from the *in-situ* measurement data. A residual is the difference between the value of measured and simulated. During the calibration process, several attempts have to be done to minimize these differences. The perfect calibrations were shown from Figure 10b, Figure 11b, Figure 12b, Figure 13b, Figure 14b, Figure 15b, Figure 16b, Figure 17b, Figure 18b, Figure 19b, Figure 20b and Figure 21b, those points are scatting less around the straight line and lying along a straight line at 45 degrees angle and the straight line has a geometric slop approximately 1.

### 5.2. Contaminant Transport Calibration

**Figure 22:**
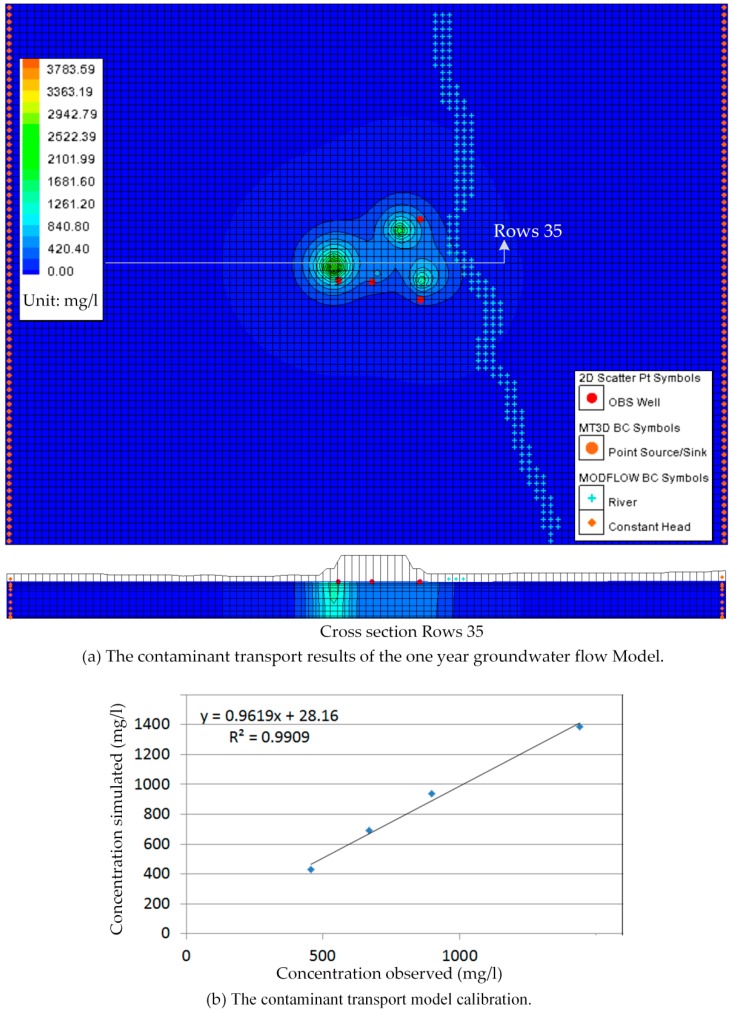
Simulation and calibration for concentration data of chlorides (November 2013 data). (**a**) The contaminant transport results of the one year groundwater flow Model; (**b**) The contaminant transport model calibration.

**Figure 23:**
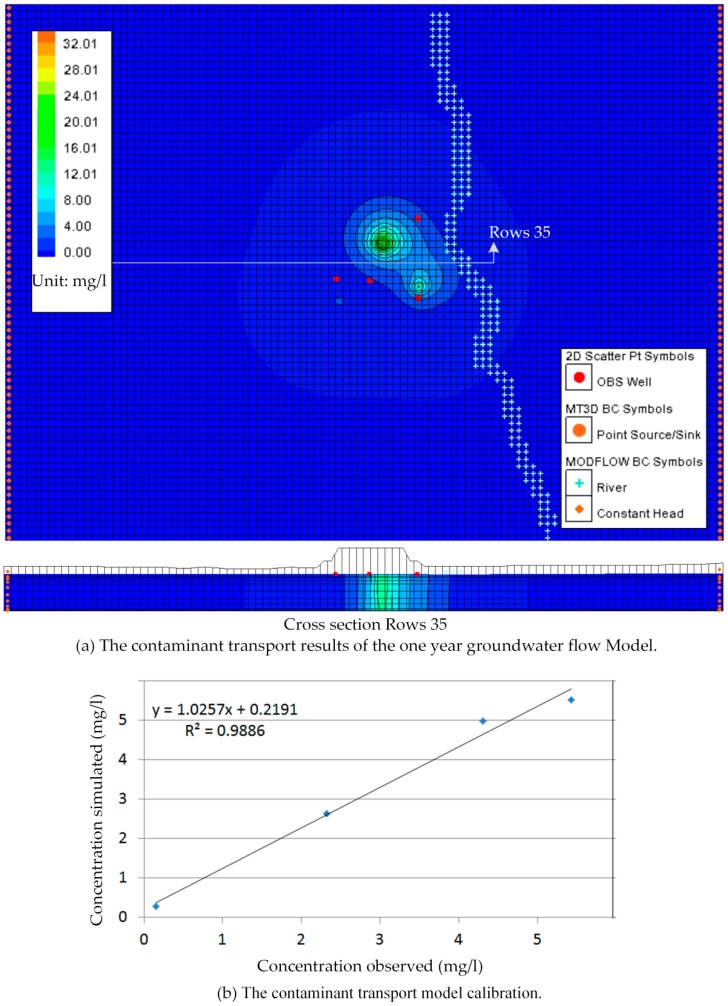
Simulation and calibration for concentration data of ammonium nitrogen (November 2013 data). (**a**) The contaminant transport results of the one year groundwater flow Model; (**b**) The contaminant transport model calibration.

The MT3DMS simulation was incorporated into the transient MODFLOW simulation model by assuming the ammonium nitrogen concentration of 35 mg/L and the chloride concentration of 4240 mg/L injected according to the one year groundwater flow model described in the transient MODFLOW simulation above. The ammonium nitrogen and chloride concentrations detected through sampling and analysis at monitoring boreholes in November 2013 listed in Table 3 were used to calibrate the contaminant transport model. The results of the contaminant transport model in November of the one year groundwater flow model were presented graphically in Figure 22 and Figure 23.

Manual adjustment and inverse modeling were also used in contaminant transport model calibration. The perfect calibrations were shown in Figure 22 and Figure 23.

### 5.3. Predicting Results

**Figure 24:**
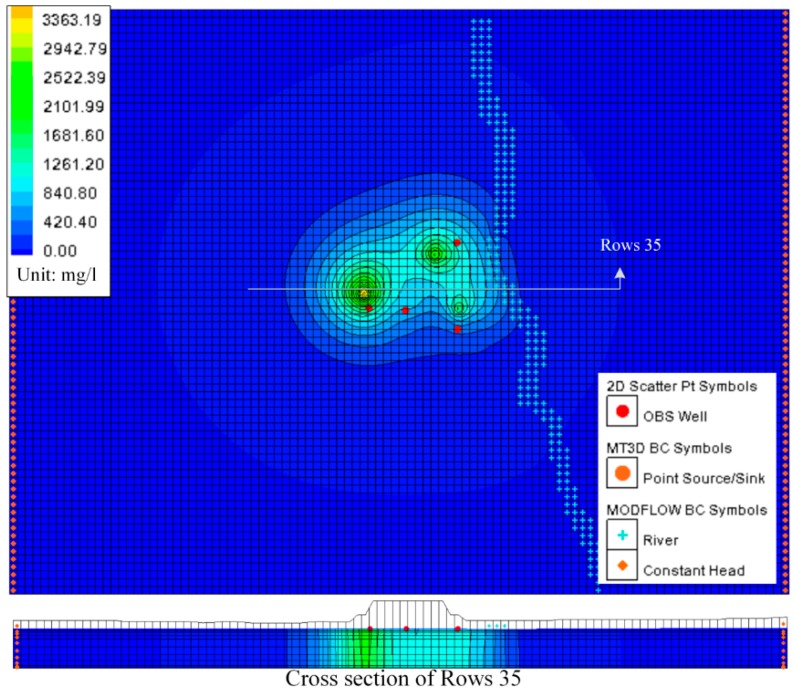
Transport predicting for chlorides after 10 years.

**Figure 25:**
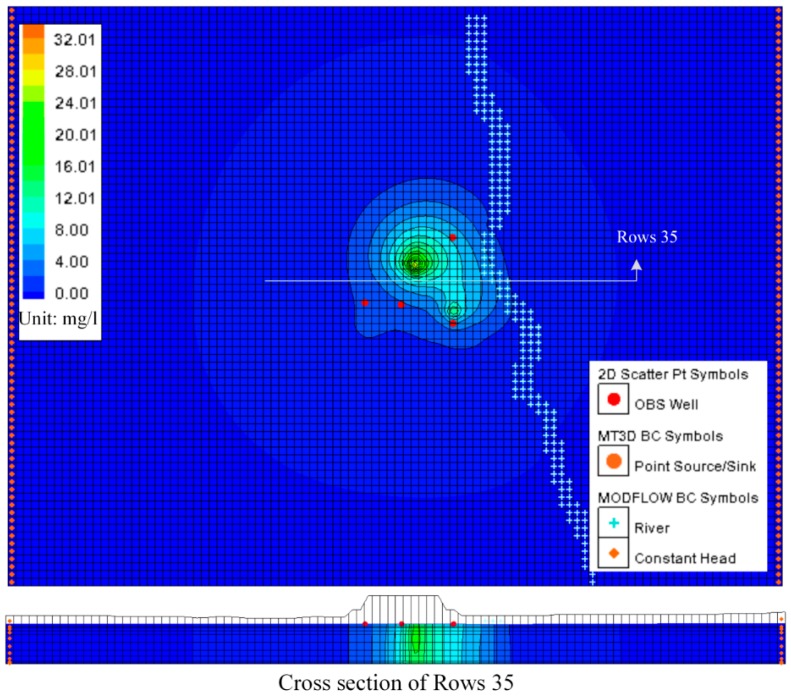
Transport predicting for ammonium nitrogen after 10 years.

After calibration, the contaminant transport model was used to predict potential plume movement 10 years into the future. In the simulations, the spreading concentration contour of ammonium nitrogen and chlorides were shown in Figure 24 and Figure 25 after 10 years. According to the simulation of the contaminant plume on 10 years, we found that the influence of groundwater flow on pollution is much more important than dispersion. Although the model appears to be quite realistic, there did not have quantitative validation to be done, because of the lack of data.

The predicting results also showed that the contamination plume has moved in the direction of the northeast (into the creek which is located in the east of the map) instead of moving along with groundwater. The results also gave a plume of contaminated groundwater that extends 80 m in length and 20 m in depth northeastward from the landfill site. The results concentration of ammonium nitrogen and chlorides in the Hsu-Hsian Creek of the Wang-Tien landfill is 467 mg/L and 3.84 mg/L, respectively, when the Monitoring Standard Value for Taiwan declared by Environmental Protection Administration (EPA in Taiwan) is 250 mg/L and 0.05 mg/L, respectively for Class A, which means that the Hsu-Hsian Creek is highly polluted and not suitable for drinking.

**Figure 26:**
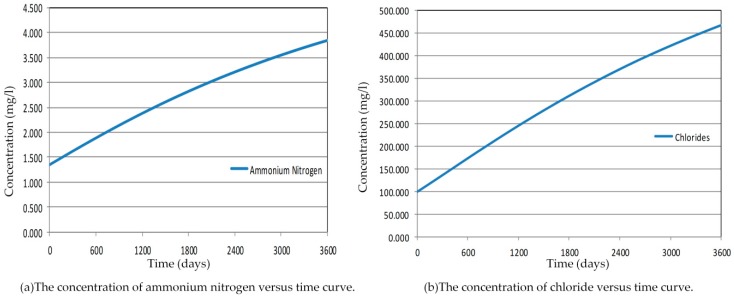
Concentration toward the Hsu-Hsian Creek with time. (**a**) The concerntration of ammonium nitrogen versus time curve; (**b**) The concerntration of chloride versus time curve.

A great number of outputs can be exported into text files. Among the text file data, all concentrations of contaminants in the aquifer for each year are available. According to the result, it shows that contaminant transport is a slow process because of sorption of pollutants in the soil layer. In 10 years, the total concentration of pollutants in the aquifer has increased by an average of 72%, including 65% of the ammonium nitrogen and 79% of the chloride. Since the Hsu-Hsian Creek is the only sink for surface flow, the entire injection of increasing concentrations from the Wang-Tien landfill site corresponds to the contaminant discharge to the Hsu-Hsian Creek. The contaminant transport toward the Hsu-Hsian Creek could be calculated by dividing the subjoint mass of contaminants with time. Figure 26 shows that the concentrations were also increasing with time. It was also expected that the all concentrations of pollutant would logically increase with time. Comparing ammonium nitrogen with chloride, the concentration of chloride were increased by 79% in 10 years which can explain that the chloride has the lowly partition coefficient$\;K_{d}$ value. The chloride is the contaminant that increases faster and with higher concentrations.

**Figure 27:**
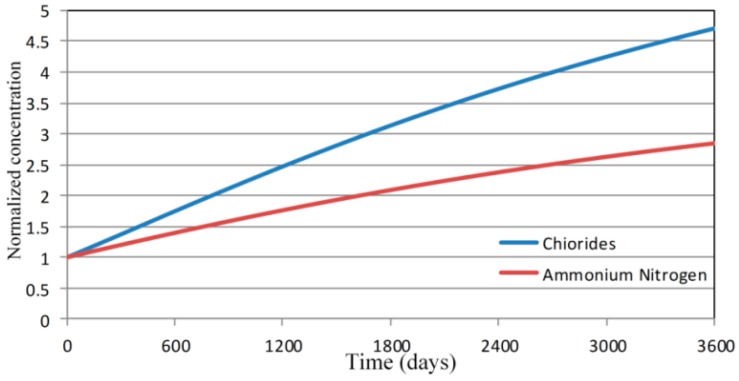
Normalized concentration toward the Hsu-Hsian Creek with time.

Both contaminant properties of the ammonium nitrogen and chlorides are entirely different, so the results of concentration need to be normalized to compare the increasing concentration difference with both. Figure 27 shows the normalized concentrations which the concentration of chloride is 1.67 times more than the concentration of ammonium nitrogen in the Hsu-Hsian Creek after 10 years.

## 6. Conclusions

GMS, a numerical calculator for simulation of groundwater flow and contaminant transport, could be used to deal with complex groundwater flow problems for contaminant transport predictions which is more convenient and suitable than analytical calculations.

In order to propose remedial solutions for a decommissioned landfill site, Wang-Tien, this study used GMS to simulate groundwater flow by incorporating the information of geology, hydrology and rainfall of the study site, and then combined contaminant transport modeling to predict ammonium nitrogen and chlorides of the site for 10 years after spreading.

According to the results of contaminant transport modeling, not only is the Wang-Tien landfill site heavily polluted both in the soil and groundwater, but also the adjacent Hsu-Hsian Creek where its concentration of chlorides is 467 mg/L (1.87 times higher than the water quality standard of EPA in Taiwan) and ammonium nitrogen is 3.84 mg/L (76.8 times higher than the water quality standard of EPA in Taiwan).

When pollutants in groundwater aquifers spread into surface water systems, the contaminant transport velocity changes its speed from slow to fast, which will spread the pollutants more quickly from the adjacent river to the whole water system and would cause irreversible damage. In the current situation, nothing has been done to the Wang-Tien landfill site where the pollution may be prolonged for centuries. In order to stop the spread of pollution and the ecosystem damage, it is obviously necessary to take some remedial actions, such as removing the whole landfill.

## Figures and Tables

**Table 1:** The hydraulic heads derived from *in-situ* borehole data monthly measured in 2013.

Borehole No.		N1 (m)	N2 (m)	N3 (m)	N4 (m)	Average (m)
Date	26/01/2013	1.9	1.706	2.216	2.198	2.005
	26/02/2013	1.76	1.986	2.016	2.048	1.9525
	26/03/2013	1.27	1.516	1.436	1.518	1.435
	26/04/2013	2.19	2.446	2.426	2.358	2.355
	27/05/2013	2.76	2.886	2.906	2.908	2.865
	26/06/2013	3.16	3.416	3.386	3.288	3.3125
	26/07/2013	3.36	3.586	3.566	3.498	3.5025
	26/08/2013	3.38	3.786	3.756	3.698	3.655
	30/09/2013	4.03	4.286	4.276	4.208	4.2
	25/10/2013	2.82	3.346	3.256	3.218	3.16
	30/11/2013	2.66	3.046	3.016	2.968	2.9225
	24/12/2013	2.545	2.956	2.861	2.813	2.79375

**Table 2:** The hydraulic conductivity data of each borehole and soil layer.

Borehole No.	Hydraulic Conductivity (m/s)
N1	1.259 × 10^−4^
N2	7.144 × 10^−5^
N3	3.079 × 10^−5^
N4	2.392 × 10^−5^
Filling material	1.26 × 10^−4^
Silty/clay	1.26 × 10^−5^
Fine sand	7.17 × 10^−4^
Clayey sand	7.17 × 10^−7^

**Table 3:** Physico-chemical parameters of water samples around the landfill in 2013.

Indicator of Water Quality	Unit	Level of Pollutants in Wells			
		N1	N2	N3	N4
Temperature	°C	25.5	25.1	26.4	25.2
pH		7.7	7.8	7.8	7.6
Electrical Conductivity	µS/cm	3990	2880	3110	5500
Total Dissolved Matters (500)	mg/L	2100	1650	1600	2850
Total Hardness as CaCO_3_ (300)	mg/L	197	174	114	276
Ammonium Nitrogen (0.05)	mg/L	4.31	5.43	2.32	0.15
Nitrite Nitrogen (0.1)	mg/L	ND	ND	ND	ND
Nitrate Nitrogen (10)	mg/L	0.14	0.10	0.01	ND
Total Organic Carbon as C (2)	mg/L	3.1	13.0	1.9	2.6
Chlorides (250)	mg/L	899	456	669	1440
Sulfate (250)	mg/L	28.9	49.2	6.64	12.6
Arsenic (0.01)	mg/L	0.0548	0.0699	0.0376	0.0336
Total Chromium (0.05)	mg/L	ND	ND	ND	ND
Copper (1.0)	mg/L	ND	<0.05	ND	ND
Manganese (0.05)	mg/L	0.08	0.04	0.05	0.08
Ferrum Iron	mg/L	<0.05	0.14	0.06	0.06
Lead (0.01)	mg/L	ND	ND	ND	<0.10
Zinc (5.0)	mg/L	<0.01	0.13	<0.01	<0.01
Nickel (0.1)	mg/L	ND	ND	ND	ND
Cadmium (0.005)	mg/L	ND	ND	ND	ND
Mercury (0.002)	mg/L	ND	ND	ND	ND

ND: Not detected and concentration lower than MDL (Method Detection Limit).

**Table 4:** Model parameters for contaminant transport modeling.

Model Parameters	Unit	Value
Effective molecular diffusion coefficient $D^{*}$	m^2^/year	0.05
Hydrodynamic dispersion coefficient $D_{xx}$*,* $D_{yy}$*,* $D_{zz}$	m^2^/year	0.0095
Longitudinal dispersivity $\alpha_{L}$	m	2.5
Transverse dispersivity $\alpha_{TH}$*,* $\alpha_{TV}$	m	0.5
Partition coefficient $K_{d}$ (Lanir *et al.* [24]) of chloride	-	0.89
Partition coefficient $K_{d}$ (Amirabdollahian and Datta, [25]) of ammonium nitrogen	-	1.17
Injection rate	m^3^/day	19.2
Maximum concentration of chlorides leachate	mg/L	4240
Maximum concentration of ammonium nitrogen leachate	mg/L	35
Porosity n For filling material layer	-	0.35
For layer silty clay		0.28
For layer fine sand		0.3
For layer clayey sand		0.04
Bulk density $\rho_{b}$ For filling material layer	g/cm^3^	1.8
For layer silty clay		1.4
For layer fine sand		1.6
For layer clayey sand		2
